# Optical Coherence Tomographic Study of a Chronically Retained Coronary Guidewire

**DOI:** 10.1155/2018/9210764

**Published:** 2018-02-26

**Authors:** Natasha Corballis, Sreekumar Sulfi, Alisdair Ryding

**Affiliations:** Department of Cardiology, Norfolk and Norwich University Hospital, Colney Lane, Norwich NR4 7UY, UK

## Abstract

Guidewire entrapment is a rare complication of coronary intervention, and management depends on the individual circumstances. This is a case of an urgent percutaneous coronary angioplasty in which a guidewire became entrapped behind a bare metal stent with subsequent fracture of the core filament, which could not be retrieved. Using optical coherence tomography, our case demonstrates extensive tissue coverage of the retained guidewire at twelve months. Five-year follow-up suggests that retained guidewires can be managed without long-term anticoagulation, even when there is substantial intra-aortic material.

## 1. Case Description

**Figure 1:**
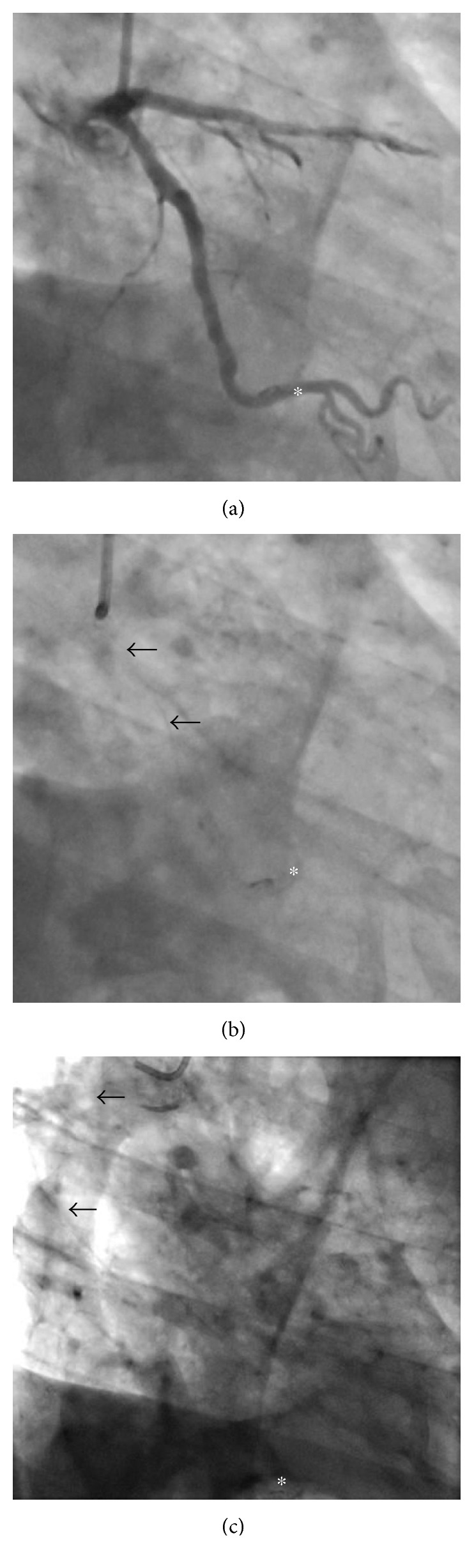
Coronary angiogram. (a) RAO 40° caudal 30° view. The radio-opaque wire tip is seen trapped behind a stent in the distal left circumflex artery (∗). (b) RAO 40° caudal 30° view. In the absence of contrast, the wire filament is seen in the proximal vessel (arrows), with the radio-opaque tip distally (∗). (c) AP view. The filament is seen in the ascending aorta (arrows), with the radio-opaque tip distally (∗).

A 76-year-old patient underwent urgent coronary angiography after presenting to hospital with a non-ST elevation myocardial infarct. Percutaneous coronary intervention (PCI) was undertaken to two severe distal stenoses in the left circumflex artery, during which the tip of the coronary guidewire (Hi-Torque Balanced Middle Weight Universal, Abbott Vascular) became entrapped behind a bare metal stent (2.5 × 12 mm Liberte, Boston Scientific). Efforts to free the tip with countertraction resulted in fracture of the guidewire core filament, which could not be retrieved. Fluoroscopy showed that the filament extended from the distal circumflex through the left main stem into the ascending aorta (Figure 1). Surgical retrieval was deemed too hazardous, and the patient was discharged on aspirin, clopidogrel, and warfarin (for 1 year).

**Figure 2:**
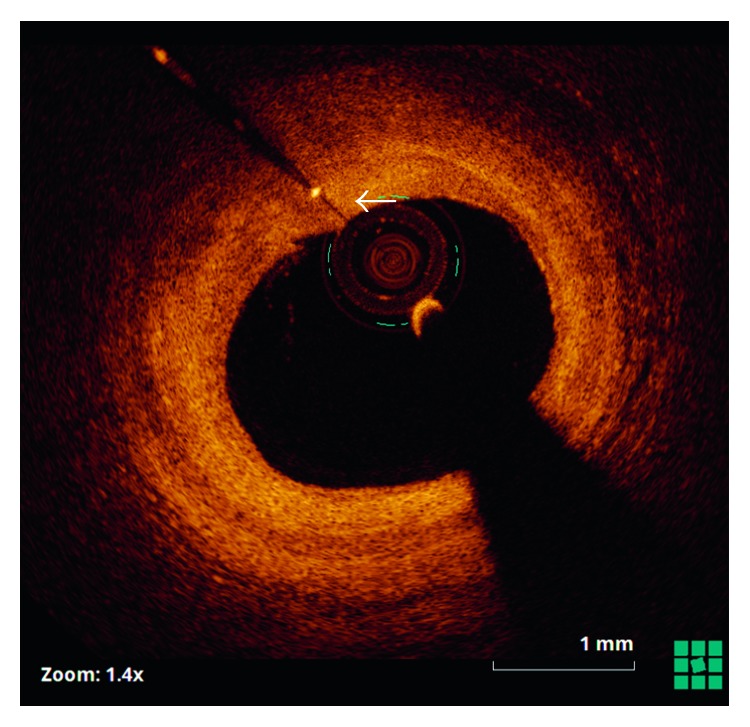
Optical coherence tomography showing the wire filament buried (arrow) within an area of intimal hyperplasia.

**Figure 3:**
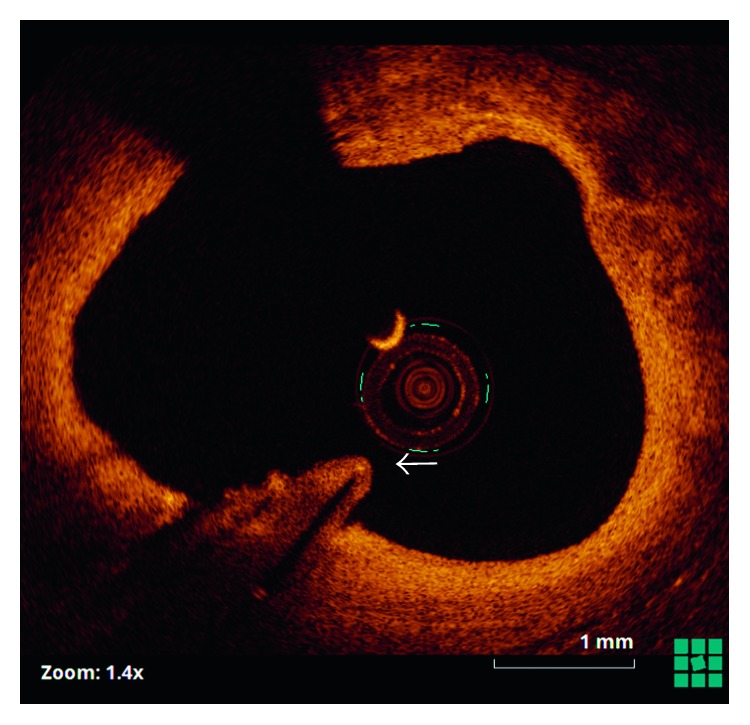
Optical coherence tomography showing the wire filament covered by a tissue bridge (arrow).

**Figure 4:**
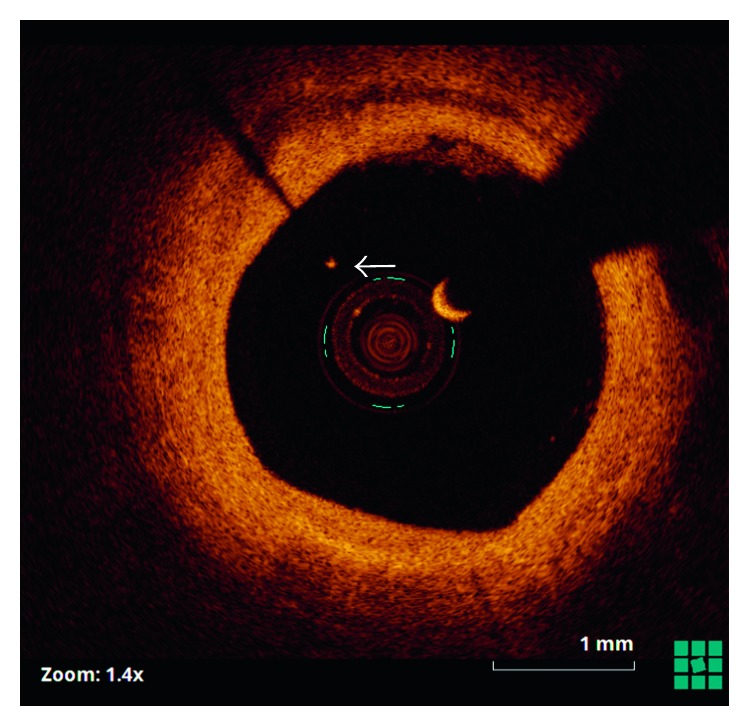
Optical coherence tomography showing the wire filament free (arrow) within the vessel lumen.

Twelve months later, angina recurred. Coronary angiography demonstrated severe diffuse restenosis within the stented segment, which was treated by balloon dilatation (Scoreflex, OrbusNeich) and a drug-coated balloon (SeQuent Please, B. Braun). Optical coherence tomography (OCT) (St. Jude, Light Lab) was then undertaken from the distal vessel back to the ostium of the left circumflex artery. This demonstrated that the retained guidewire filament was covered by neointimal hyperplasia over approximately 50% of its length (Figure 2), with evidence of tissue bridges (Figure 3). The remainder of the wire remained free within the vessel lumen with no evidence of tissue coverage (Figure 4).

Five years after guidewire entrapment, the patient has remained free of thrombotic complications on aspirin and clopidogrel.

## 2. Discussion

Guidewire entrapment is a very rare complication of PCI (occurring in 0.08%) [1]. The most common mechanisms for fracture of the coronary guidewire are entrapment, excessive rotation, or forceful traction of the guidewire [2]. The risk of guidewire entrapment and fracture is therefore dependent on two factors: the first is lesion characteristics, and the second is the type of guidewire used. A tortuous, calcified, and distal lesion is more likely to cause fracture due to the risk of overcoiling and bending of the guidewire.

Regarding the guidewire type, a polymer-jacketed guidewire (as in this case) has been implicated as a risk factor (occurring in 35% of the reported cases) [3]. The site of fracture is most likely to occur at the junction point between the 3 cm flexible tip and the remainder of the guidewire [4]. The retention of a long filament, as in this case, is therefore unusual.

The need for surgical removal and the urgency of this depend on individual circumstances, in particular the immediate ischaemic/haemodynamic complications [5]. Some authors suggest that the entrapped material need not be surgically removed if it is in the distal coronary bed, but it is necessary if there is protrusion into the ascending aorta [5]. Surgical removal can be difficult and may lead to further vessel damage [6]. This OCT study provides a unique insight into the medium-term vascular response to guidewire entrapment. We found that a substantial proportion of the intracoronary wire was covered by tissue, particularly where it was adjacent to the vessel wall. In addition, tissue bridges were seen to extend from the wall into the lumen along the wire. The cellular components of the tissue are unknown, but probably resemble neointimal hyperplasia. We do not know whether this process has continued in the longer term, nor whether it has also occurred in the aortic portion of the wire. Tissue coverage of the metal filament is likely to have minimised the risk of thrombotic complications.

This case has two main implications: firstly, guidewire entrapment can be managed conservatively with good long-term outcomes, even when there is a material within the ascending aorta. Secondly, long-term anticoagulation is not required, and dual antiplatelet therapy appears to be sufficient.
